# Left superior vena cava's unconventional path to left atrium drainage: A case report

**DOI:** 10.1016/j.radcr.2024.06.089

**Published:** 2024-07-26

**Authors:** Muhammad Idrees, Waleed Tariq, Rashid Asghar, Muhammad Junaid Tahir, Khabab Abbasher Hussien Mohamed Ahmed, Zohaib Yousaf

**Affiliations:** a Multan Institute of Kidney Diseases, Multan, Pakistan; b Mayo Hospital, Lahore, Pakistan; c Shaukat Khanum Memorial Cancer Hospital and Research Center, Lahore, Pakistan; d Faculty of Medicine, University of Khartoum, Khartoum, Sudan; e Tower Health, Reading, PA, USA

**Keywords:** Persistent left superior vena cava, Left atrium, Central venous catheterization, Chest X-ray, End-stage renal disease

## Abstract

Persistent left superior vena cava (PLSVC) is a rare congenital anomaly. We presented PLSVC in a patient with end-stage renal disease (ESRD) requiring hemodialysis. The left internal jugular vein was utilized for central venous access due to difficult central vascular access, resulting in a diagnosis of PLSVC draining in the left atrium. This case underscores the importance of awareness of anatomical variations before central catheter placement.

## Introduction

Arteriovenous (AV) access is the preferred route for hemodialysis in end-stage renal disease (ESRD) [1]. A central venous catheter (CVC) is commonly used for temporary vascular access in patients requiring urgent HD. Due to direct access to the superior vena cava (SVC) and right atrium, the right internal jugular vein is the preferred site for CVC placement over the left side and the subclavian veins [2].

Embryologically, anterior and posterior cardinal veins carry deoxygenated blood from the cranial and caudal parts of the embryo, respectively. During the eighth week of development, the anastomosis between the left and right anterior cardinal veins forms the left brachiocephalic vein, shunting blood from left to right. Post-anastomosis, the proximal segment of the right anterior and common cardinal veins evolves into SVC. In contrast, the left anterior and common cardinal veins obliterate with the formation of the ligament of Marshall and coronary sinus, respectively. The failure to develop the ligament of Marshall can result in PLSVC formation [3,4]. PLSVC is the most common thoracic venous anomaly, with a prevalence of 0.2% to 3% in the general population and 1.3% to 11 % in the population with an associated cardiac anomaly. Most cases are discovered incidentally during CVC placement or imaging-like chest computed tomography [5]. The ideal site for CVC placement in the presence of PLSVC is unclear [6]. We reported PLSVC identified in a patient with multi-access failure. Access was successfully established through the left internal jugular vein.

## Case presentation

A 43-year-old female with a history of ESRD on maintenance HD for the last year presented to the emergency department with progressive shortness of breath and anuria over the previous 4 days. She had an unrestricted intake of fluids for 1 week due to a hot summer. Previously, she had 2 HD sessions per week instead of the recommended 3 sessions per week due to financial constraints. The patient had opted for no arteriovenous (AV) fistula and had had HD sessions from indwelling CVC at various sites. She had her last session of HD 1 week ago from CVC in the right internal jugular vein. Her CVC was removed 1 week before presentation due to concern for central line-associated bloodstream infection (CLABSI) with a plan for tunneled catheter placement after treatment of active infection.

Upon presentation, she was tachycardiac (110 beats per minute), hypertensive (210/100 mmHg), tachypneic (31 breaths per minute), and desaturated to 76% on room air. Examination revealed periorbital oedema and grade III pedal oedema extending up to the shins bilaterally, and crackles were audible all over the chest. On chest X-ray (CXR), broncho-vascular markings, septal thickening, and ground glass appearance were prominent concerning pulmonary oedema. The patient's renal function showed high levels of urea and creatinine, i.e., 121 mg/dL and 9.28 mg/dL, respectively. A working diagnosis of hypertensive emergency with pulmonary oedema secondary to inadequate renal replacement therapy was made, and the patient was planned for urgent hemodialysis.

**Fig. 1:**
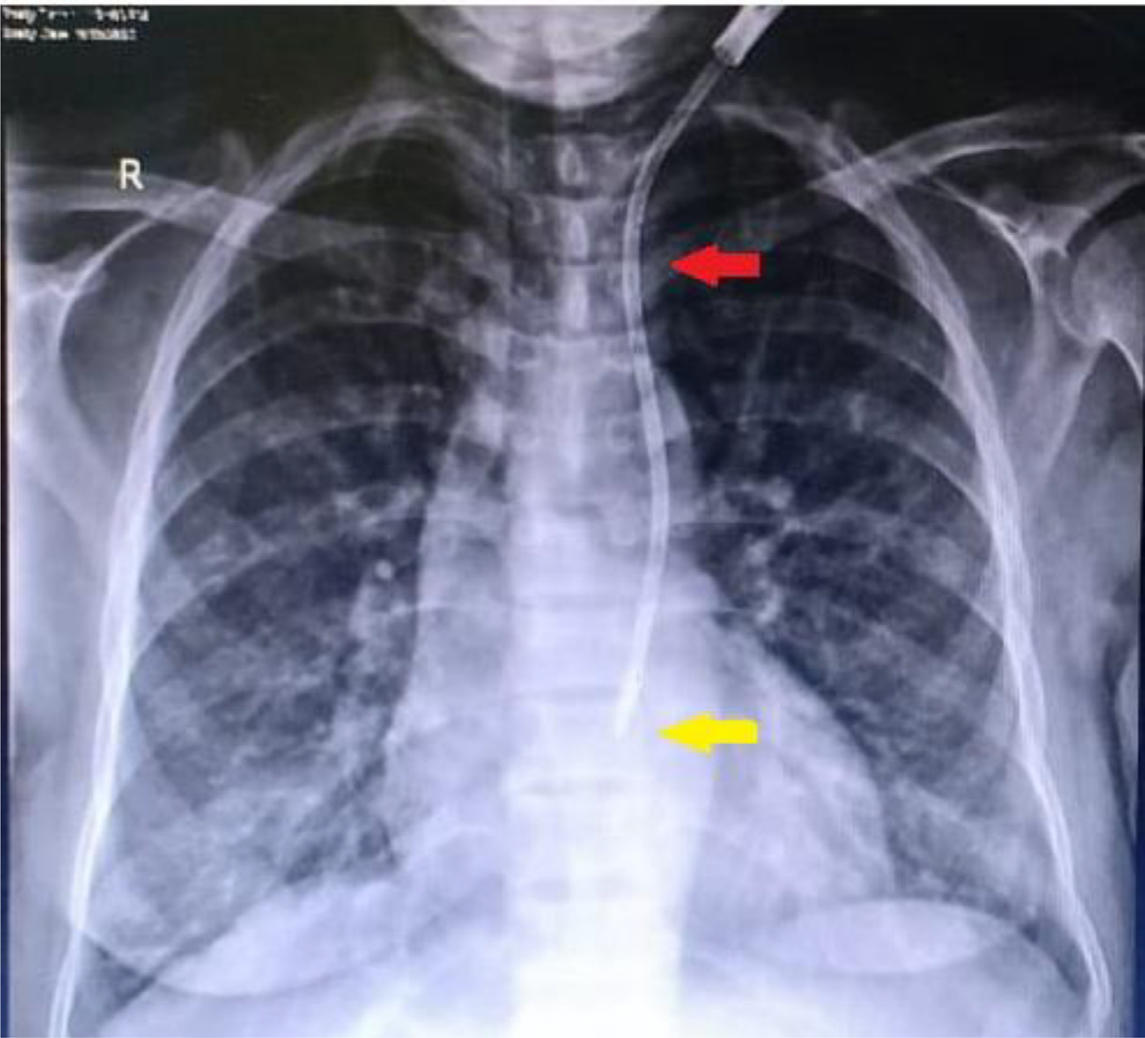
Posterior to anterior (PA) view of post-CVC insertion chest X-ray (Red arrow pointing at the lumen of CVC, Yellow arrow pointing at the tip of CVC).

**Fig. 2:**
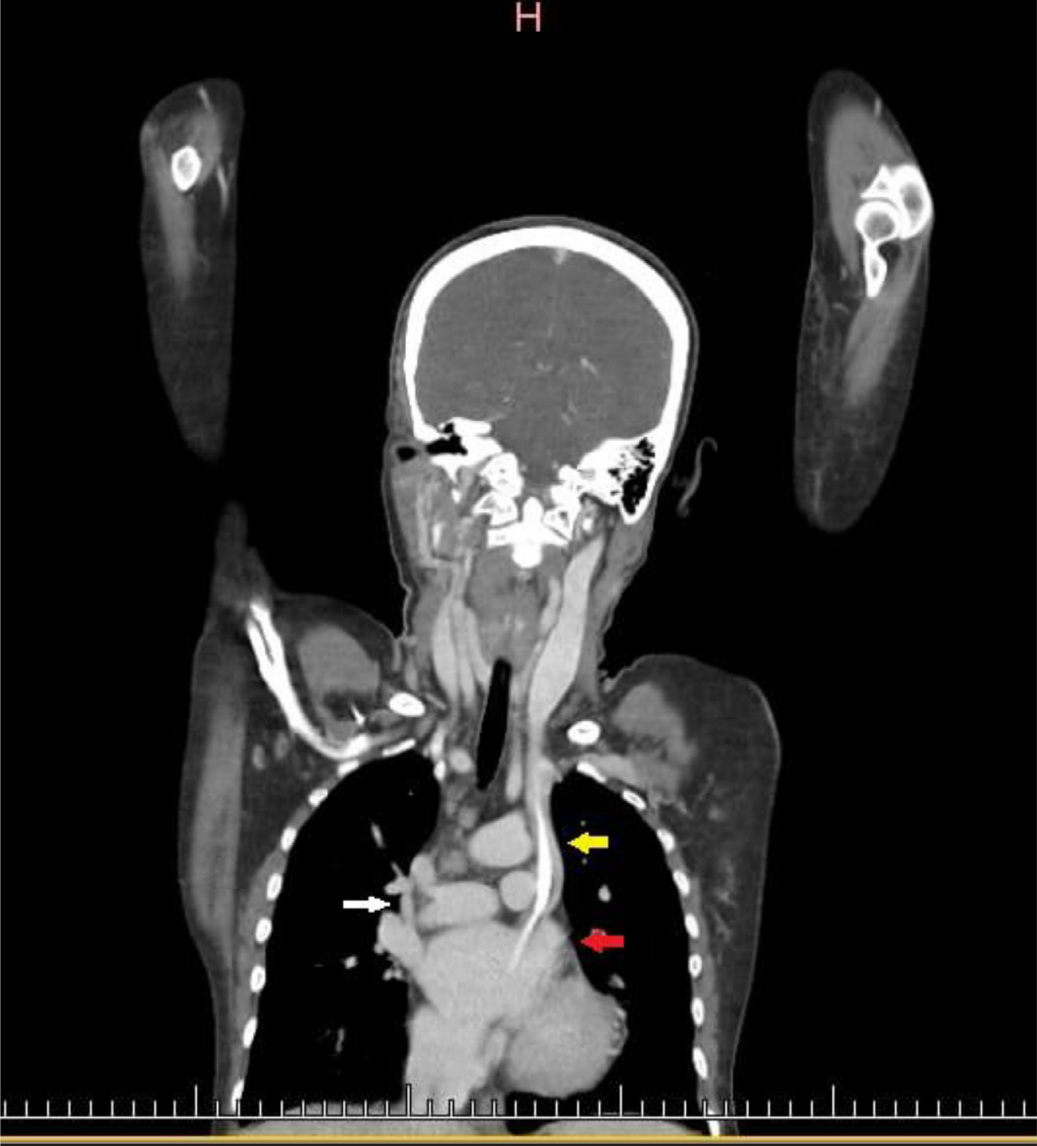
Coronal view of CT scan (White arrow pointing at right SVC, Yellow arrow pointing at PLSVC, Red arrow pointing at left atrium).

**Fig. 3:**
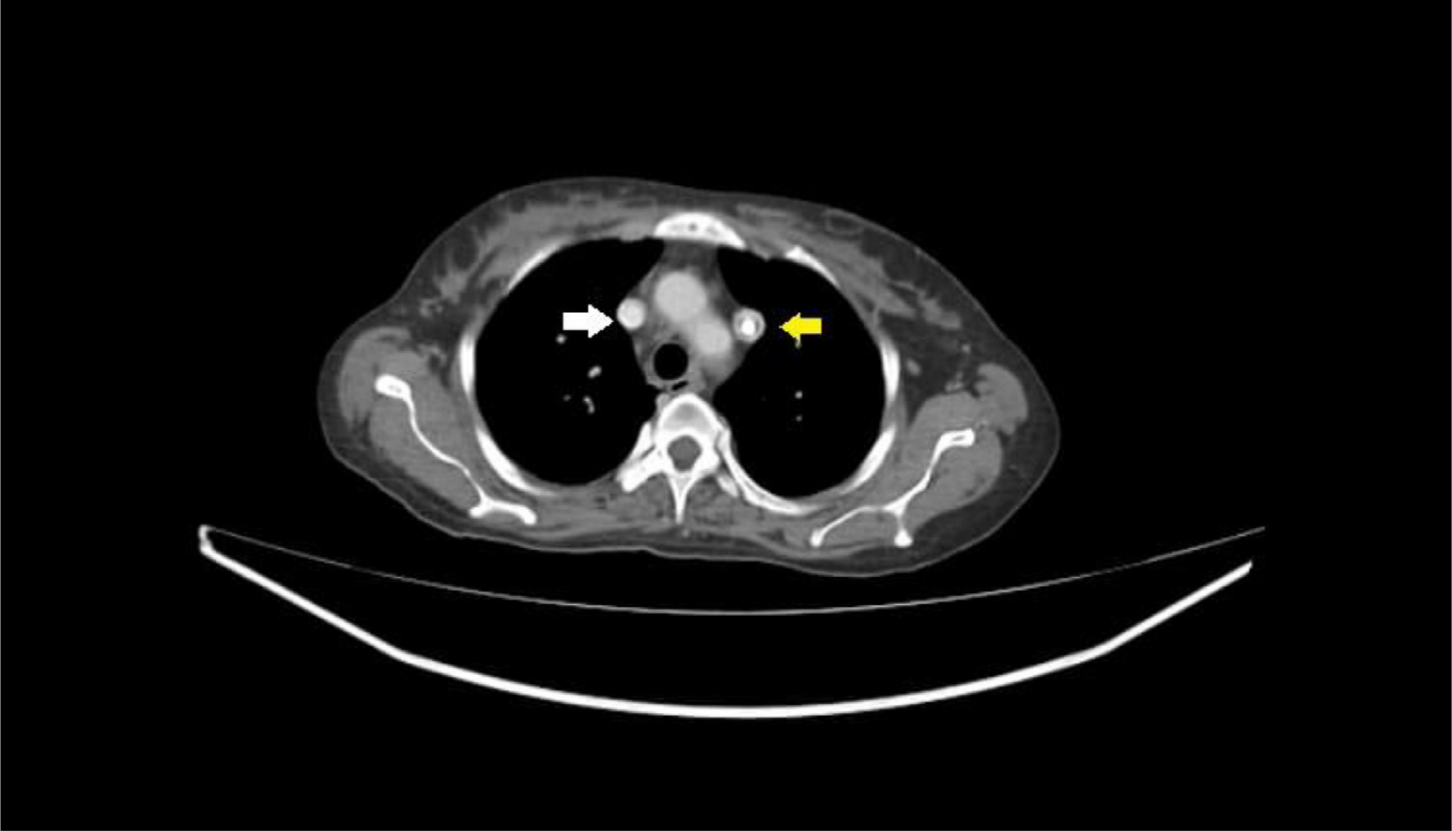
Axial view of CT scan (Yellow arrow pointing at PLSVC, white arrow pointing at right SVC).

A point-of-care ultrasound revealed stenosis of the right jugular, bilateral femoral, and subclavian veins, likely secondary to the multiple CVC insertions over the last year. CVC was inserted in the left internal jugular vein for urgent HD. A post-CVC-insertion CXR disclosed an abnormal position of the dialysis catheter tip along the left mediastinal border (Fig. 1). A computed tomography angiography (CTA) showed PLSVC draining into the left atrium following the path along the left sternal border (Fig. 2, Fig. 3). A transthoracic echocardiogram was unrevealing for any other concurrent cardiac anatomic anomalies. The rest of the hospital stay was uncomplicated. The patient underwent surgery for AV fistula formation and continued to receive hemodialysis sessions through CVC in PLSVC without any complications.

## Discussion

The first case of persistent left SVC was reported in 1787, a common congenital venous circulation anomaly [7]. Lim and H'ng had also reported a PLSVC diagnosed during CVC catheterization in the left internal jugular vein for HD and later confirmed on CTA. HD was done for 5 months without complications [2]. Jang et al. also reported a left CVC tip seen along the left mediastinal border on chest radiography. CTA led to a diagnosis of PLSVC [8].

An opaque line along the medial border of the mediastinum in post-CVC chest X-ray strongly suggests suspicion of PLSVC which must be confirmed by further radiologic investigation. Various imaging modalities have been implied for in-depth confirmation, especially contrast-enhanced computed angiogram, magnetic resonance venography, and conventional venography [9]. After finding a catheter along the medial border of the mediastinum, we performed CTA to explore the tract. We found contrast enhancement on the left side lateral to the aortic arch (Fig. 1, Fig. 2) and draining into the left atrium (Fig. 1), confirming the presence of PLSVC. At the same time, the contrast-enhanced tract was also illustrated on the right side draining into the right heart which was the right SVC (Fig. 1, Fig. 2). Sonavane, Sushilkumar K., et al. and Azizova, Aynur, et al. reported similar radiologic findings on cross-sectional and axial views [10,11].

Hana et al. reported a PLSVC draining through the left brachiocephalic vein in the left atrium posteriorly [12]. This finding is similar to our case. PLSVC draining into the left atrium is the cause of right to left shunt and is usually associated with cardiac anomalies. However, this can be an isolated finding with no other cardiac anomalies [13].

Association of various cardiac anomalies including ventricular septal defects, atrial septal defects, or tetralogy of Fallot with PLSVC must be screened with transthoracic echocardiography or cardiac MRI to avoid mal-positioning of CVC [14]. Patients with PLSVC draining into the left atrium may develop a large right-to-left shunt. Among such patients, dialysis catheters must be avoided and the use of the femoral vein is recommended. The precatheter placement echocardiography is advised to avoid any complications. Similarly, femoral access must be used again in patients with PLSVC with absent RSVC.

PLSVC is most commonly diagnosed incidentally on post-CVC chest radiographs [15]. Use of the left internal jugular vein, as in our case, led to an incidental diagnosis of PLSVC. Authors suggest close hemodynamic monitoring due to a risk of arrhythmias, embolization, shock, and cardiac arrest associated with the placement of CVC in PLSVC [16]. An echocardiogram may be helpful to evaluate for concurrent cardiac abnormalities.

By European Renal Best Practice (ERBP) guidelines [17], the right internal jugular vein is the access of choice among patients requiring initiation of urgent Hemodialysis as there is minimal risk of infection if inserted under strict aseptic techniques. The next approach could be adopted via the left internal vein but the subclavian vein is not recommended as the first choice among such patients as there is an increased risk of venous stenosis. In addition to the higher incidence of thrombosis, there is a higher chance of infection and bacteremia among patients using the femoral as an access site for hemodialysis, that's the reason it is not recommended at any cost among patients needing urgent hemodialysis.

The gold standard mode for Hemodialysis for maintenance hemodialysis is Arteriovenous fistula, followed by arteriovenous graft and then tunneled catheter at least. For the patients requiring chronic renal replacement therapy via hemodialysis with a history of catheter infection, the recommendations by the Infectious Disease Society of America (IDSA) must be followed. In case of evident infection of the catheter insertion site without pyrexia, topical antibiotics must be applied. The local site infection resistant to topical therapy must be treated with systemic antimicrobials. Catheter removal is only advised when both steps fail to attain resolution. When catheter-related bloodstream infection (CRBSI) is confirmed by either clinical signs of active disease (fever, tachycardia, chills, or hypotension during dialysis) or sepsis or sepsis-related complications like thrombophlebitis and metastatic infection appear, the catheter must be removed with immediate initiation of empirical antibiotic therapy after sending blood cultures. The patients facing limited access issues with existent CRBSI, remove the catheter only when other potential insertion sites are available. At this time, catheter removal followed by systemic antibiotic therapy and re-insertion after 48-72 hours is recommended. In the absence of another site for re-insertion, guide wire-assisted catheter exchange is advised after 48-72 hours of systemic antibiotic therapy. Accepting the problems of high failure rate, venous sclerosis, and stenosis of guidewire assisted exchange, keep the catheter in situ and start catheter salvage with antibiotic lock. If this fails then remove the catheter. After all, re-consider AVF, Arteriovenous graft, or move towards peritoneal dialysis [18].

## Conclusion

PLSVC is usually an asymptomatic, incidentally discovered congenital anomaly. Right heart access through the left subclavian vein in the case of PLSVC is challenging but possible. Close hemodynamic monitoring and an echocardiogram are prudent in the case of using a CVC inserted through a PLSVC.

## Author contributions

MI and RA conceived and designed the case report, and were responsible for data collection and acquisition of data. MI, WT, ZY, and MJT performed the literature review and wrote the manuscript. K.A.H.M.A., ZY and MJT reviewed and critically revised the manuscript. All authors have approved the final manuscript.

## Ethical approval

Ethical approval was not required because this is a case report.

## Patient consent

Written informed consent was obtained from the patient for publication and any accompanying images. A copy of the written consent is available for review by the Editor-in-Chief of this journal on request. All patient data were de-identified to maintain confidentiality.
